# Comparative proteomic analysis of mitochondria isolated from *Euglena gracilis* under aerobic and hypoxic conditions

**DOI:** 10.1371/journal.pone.0227226

**Published:** 2019-12-31

**Authors:** Shun Tamaki, Kohei Nishino, Takahisa Ogawa, Takanori Maruta, Yoshihiro Sawa, Kazuharu Arakawa, Takahiro Ishikawa

**Affiliations:** 1 Institute of Agricultural and Life Sciences, Academic Assembly, Shimane University, Matsue, Shimane, Japan; 2 Core Research for Evolutional Science and Technology (CREST), Japan Science and Technology Agency (JST), Chiyoda-ku, Tokyo, Japan; 3 Institute for Advanced Biosciences, Keio University, Tsuruoka, Yamagata, Japan; 4 Systems Biology Program, Graduate School of Media and Governance, Keio University, Fujisawa, Kanagawa, Japan; Universite Paris-Sud, FRANCE

## Abstract

The unicellular microalga *Euglena gracilis* produces wax esters for ATP acquisition under low-oxygen conditions. The regulatory mechanism of wax ester production is not yet understood. Indeed, our previous transcriptomic analysis showed that transcript levels of genes involved in the wax ester synthesis hardly changed under hypoxic conditions, suggesting contribution of post-transcriptional regulation. In this study, we conducted a proteome analysis of *E*. *gracilis* mitochondria, as this organelle employs the fatty-acid synthesis pathway under hypoxic conditions. Mitochondria were isolated from *E*. *gracilis* SM-ZK strain treated with both aerobic and hypoxic conditions and used for shotgun proteomic analysis. Three independent proteomic analyses succeeded in identifying a total of 714 non-redundant proteins. Of these, 229 were detected in common to all experiments, and 116 were significantly recognized as differentially expressed proteins. GO enrichment analysis suggested dynamic changes in mitochondrial metabolic pathways and redox reactions under aerobic and hypoxic conditions. Protein levels of bifunctional enzymes isocitrate lyase and malate synthase in glyoxylate cycle were 1.35-fold higher under hypoxic conditions. Abundances of the propionyl-CoA synthetic enzymes, succinyl-CoA synthetase and propionyl-CoA carboxylase, were also 1.35- and 1.47-fold higher, respectively, under hypoxic conditions. Protein levels of pyruvate:NADP^+^ oxidoreductase, a key enzyme for anaerobic synthesis of acetyl-CoA, which serves as a C2 donor for fatty acids, showed a 1.68-fold increase under hypoxic conditions, whereas those of pyruvate dehydrogenase subunits showed a 0.77–0.81-fold decrease. Protein levels of the fatty-acid synthesis enzymes, 3-ketoacyl-CoA thiolase isoforms (KAT1 and KAT2), 3-hydroxyacyl-CoA dehydrogenases, and acyl-CoA dehydrogenase were up-regulated by 1.20- to 1.42-fold in response to hypoxic treatment. Overall, our proteomic analysis revealed that wax ester synthesis-related enzymes are up-regulated at the protein level post-transcriptionally to promote wax ester production in *E*. *gracilis* under low-oxygen conditions.

## Introduction

*Euglena gracilis* is a motile unicellular flagellate capable of photosynthesis and has been proposed as a biocatalyst for the production of bioenergy and various valuable compounds [1]. *E*. *gracilis* cells have the ability to synthesize a large amount of the storage polysaccharide β-1,3-glucan, known as “paramylon”, under aerobic conditions. These cells accumulate up to 50% (on a dry weight basis) of paramylon [2]. However, upon cell culture under low-oxygen conditions, the paramylon is degraded and converted into wax esters consisting of saturated fatty acids and alcohols with C14 chains as the major species. This phenomenon has been designated as wax-ester fermentation due to the generation of ATP without net ATP loss during the production of wax esters [3,4]. Myristic acid (C14:0) has more potential as an alternative bio-jet fuel than medium-length fatty acids produced by other algae, such as palmitic acid (C16:0) and stearic acid (C18:0), because myristic acid has a low freezing point and an adequate Cetane number (66.2) [5].

In *E*. *gracilis* mitochondrial wax-ester synthesis, fatty acids are synthesized by a reversal of β-oxidation process [6]. Some of the metabolic enzymes involved in this process have been biochemically analyzed. Acetyl-CoA stemming from pyruvate by a peculiar oxygen-sensitive enzyme, pyruvate:NADP^+^ oxidoreductase (PNO), serves as a primer and a C2 donor [7,8]. Synthesis of odd-numbered fatty acids starts from propionyl-CoA, which is synthesized *via* the methylmalonyl-CoA pathway. Fatty acid synthesis then proceeds *via* a condensing step catalyzed by 3-ketoacyl-CoA thiolase (KAT) [9]. Acyl-CoA was believed to be generated from enoyl-CoA by the catalysis of *trans*-2-enoyl CoA reductase (TER) [10] and then exported from mitochondria; however, the substantial contribution of TER enzyme to fatty acid synthesis remains unclear [11]. Acyl-CoA and the fatty alcohol subsequently generated by a fatty acyl-CoA reductase are then esterified by wax synthase/diacylglycerol acyltransferase to produce wax esters in the microsomes [12,13].

In addition to wax esters, *E*. *gracilis* also produces a large amount of succinate, a useful material for the production of bioplastics, under anaerobic conditions. A previous study suggested that *E*. *gracilis* produces succinate *via* the reductive TCA cycle in mitochondria [14]. Thus, the metabolic flux shift in mitochondria in response to low-oxygen conditions is an important process to produce valuable compounds in *E*. *gracilis*, such as wax esters and succinate.

Aiming to elucidate the regulatory mechanism of hypoxic wax ester synthesis, we previously performed a comparative transcriptomic analysis of *E*. *gracilis* and found that most genes differentially expressed under low-oxygen conditions are related to photosynthesis, nucleotide metabolism, oxidative phosphorylation, and fatty acid metabolism, but not to wax ester synthesis [15]. Thus, the metabolic regulation of wax ester production in response to low-oxygen conditions is likely to occur at the post-transcriptional level. Herein, we focused on analyzing the mitochondrial proteome and performed a shotgun proteomic analysis of mitochondria isolated from *E*. *gracilis* cells under aerobic and hypoxic conditions to understand the metabolic regulatory mechanism during wax ester synthesis in *E*. *gracilis*.

## Materials and methods

### Strain and culture

*E*. *gracilis* strain SM-ZK (non-photosynthetic mutant) was grown in Koren–Hutner (KH) medium [16] under continuous light conditions (50 μmol m^−2^ s^−1^) at 26°C with rotary shaking (120 rpm). For aerobic treatment, cells cultured for 6 d were aerated with aseptic air for an additional 24 h. On the other hand, for hypoxic treatment, 6 d-cultures were completely sealed and left to stand still for 24 h without shaking after the replacement of air with nitrogen gas. Notably, cell numbers did not change during additional 24 h aerobic or hypoxic treatments, because the 6 d-cultures have reached to the stationary phase.

### Mitochondria isolation from *E*. *gracilis*

*E*. *gracilis* cells were collected and resuspended in lysis buffer (25 mM HEPES-KOH, pH 7.5, 0.25 M sucrose and 0.5 mM EDTA) and partially disrupted by gas nebulizer-mediated collision using BioNeb disruption system (Glas-Col, Terre Haute, IN). After centrifugation at 1,000 × *g* for 5 min at 4°C, the supernatants were centrifuged at 12,000 × *g* for 10 min at 4°C. Precipitations were resuspended in the lysis buffer and mitochondrial fractions were purified by step-wise Percoll density (10, 30, and 50%) centrifugation at 9,000 × *g* for 40 min at 4°C. The purified mitochondrial fractions were recovered at the interface of 30% and 50% Percoll. After centrifugation at 13,000 × *g* for 10 min at 4°C, supernatants were removed to collect the mitochondria pellets ready for use. The extent of non-mitochondrial contaminants such as plastids were estimated very low (1% or less) by calculating with normalized abundances of some representative proteins.

### Peptide preparation

*E*. *gracilis* mitochondrial fractions were resuspended in extraction buffer (50 mM Tris-HCl, pH 7.5, 10% glycerol, 1 mM 6-aminocapric acid, 1.3 mM benzamidine, 2 mM dithiothreitol, 1 mM phenylmethylsulfonyl fluoride, and 10 mg/mL polyvinylpyrrolidone) and disrupted by sonication. The mitochondria homogenates were centrifuged at 13,000 × *g* for 15 min at 4°C, and the supernatant was collected. Protein contents were determined by the Bradford method [17]. Protein extracts containing 150 μg of proteins were purified by a methanol/chloroform precipitation method. Protein extracts were dissolved in 8 M urea, diluted to 800 μL with 50 mM NH_4_HCO_3_, and, then, reduced with 5 mM dithiothreitol for 30 min at 60°C. These steps were followed by alkylation with 0.74 mM iodoacetamide at room temperature for 30 min. Finally, the reduced and alkylated protein solutions were incubated with 5 μg of trypsin overnight at 37°C. The reaction was stopped with 0.57% trifluoroacetic acid. The resulting peptide solutions were desalted and concentrated using MonoSpin C18 (GL Sciences, Tokyo, Japan). Peptide solutions were then subjected to fractionation in Agilent 3100 OFFGEL Fractionator (Agilent Technologies, Santa Clara, CA), desalted, and concentrated using GL-Tip SDB (GL Sciences). The final treated samples were dissolved in a solution containing 0.1% formic acid and 5% acetonitrile and used for mass spectrometry.

### LC-MS/MS analysis

LC-MS/MS analysis was performed using a SYNAPT G2 mass spectrometer (Waters, Milford, MA) interfaced with a nanoAcquity UPLC (Waters). Peptide samples were loaded onto a nanoACQUITY UPLC Symmetry C18 Trap Column (180 μm i.d. × 20 mm, 5 μm, Waters), washed, and placed in line with an ACQUITY UPLC BEH C18 Column (130Å, 1.7 μm, 2.1 mm × 50 mm, Waters). Peptides were eluted from the second-dimension column with a linear gradient from 99% of H_2_O/formic acid (99.9:0.1, v/v) (buffer A) to 45% of acetonitrile/formic acid (99.9:0.1, v/v) (buffer B) for 100 min at a flow rate of 300 nL/min. Leucine enkephalin (556.277 Da) was used for the detector set up. Mass (m/z) calibration was performed on a separate infusion of [Glu1]-fibrinopeptide B (785.843 Da). Other parameters included a capillary voltage of 2.5 kV, sample cone of 50 V, extraction cone of 2.8 V, source temperature at 80°C, cone gas flow of 50 l/h and nano flow gas of 0–0.6 bar, and purge gas of 0–300 l/h.

### Data processing, searching, and analysis

The progenesis QI software (Waters Ltd./Nonlinear Dynamics, Newcastle upon Tyne, UK) was used to analyze the raw data files imported from the Synapt G2 mass spectrometer. Each run in the experiment was shown as an ion intensity map, which was representative of the sample MS signal in terms of m/z and retention time. To combine and compare results from different runs, the Progenesis QI aligned the data to compensate for the between-run variation during chromatography. Processed data were analyzed using trypsin as the cleavage protease, one missed cleavage was allowed; fixed modification was set to carbamidomethylation of cysteines, and variable modification was set to oxidation of methionine. Minimum identification criteria included 1 fragment ions per peptide, 2 fragment ions per protein, and a minimum of 1 peptide per protein. Peptide identification was performed using the identity algorithm implemented in the Progenesis QI by searching against *E*. *gracilis* protein database constructed by translating cDNA sequences having high BUSCO completeness (91.09%) [15]. False discovery rates (FDR) threshold for peptide and protein identification were set at 4%. To quantify the relative abundance of identified proteins, each run was automatically normalized using an ensemble data set constructed from the aligned runs as recommended by the Progenesis QI software manual. Protein abundance is then calculated from the sum of all unique normalized peptide ion abundances for a specific protein on each run. Only diagnostic peptides were used for quantification at the protein level, and data on abundance changes between aerobic and hypoxic treatments are provided after using ANOVA statistical significance (*p*<0.05). The scoring algorithm implemented in Progenesis software was used to determine confidence values. To show changes in the abundance of each protein under aerobic and hypoxic conditions, we compared maximum fold change values in peptide counts. Proteins with significantly different abundances (ANOVA: *p*<0.05) between aerobic and hypoxic conditions were defined as differentially expressed proteins (DEPs). Gene ontologies (GOs) of identified proteins for the categories of biological process, molecular function and cellular compartment were identified as described previously [15].

## Results and discussion

### Protein identification and pathway analysis

In this study, we used SM-ZK strain, an *E*. *gracilis* non-photosynthetic mutant, which has been used for many studies so far to understand physiological and biochemical functions on wax ester fermentation including mitochondrial metabolic enzymes [e.g., 3, 9]. Previously, we have shown that the *E*. *gracilis* cells accumulate significant amount of wax esters after 24 h low-oxygen treatment [11, 13], indicating that the metabolic enzymes related to wax-ester production can be expected to fully express in the cells by the treatment. Thus, heterotrophically grown *E*. *gracilis* SM-ZK cells were treated with aerobic or hypoxic conditions for 24 h, and intact mitochondria were isolated from each treatment. After digestion of soluble proteins extracted from mitochondria, nanoLC/MS/MS analysis, protein identification, and quantification were performed. Three independent experiments identified total 714 proteins without duplication. Among them, 229 proteins were detected through all three independent experiments and 116 proteins were identified as differentially expressed proteins (DEPs) with significant differences (ANOVA: *p*<0.05) (S1 Table). Of the 116 DEPs identified, 60 were upregulated under hypoxic conditions.

**Fig 1:**
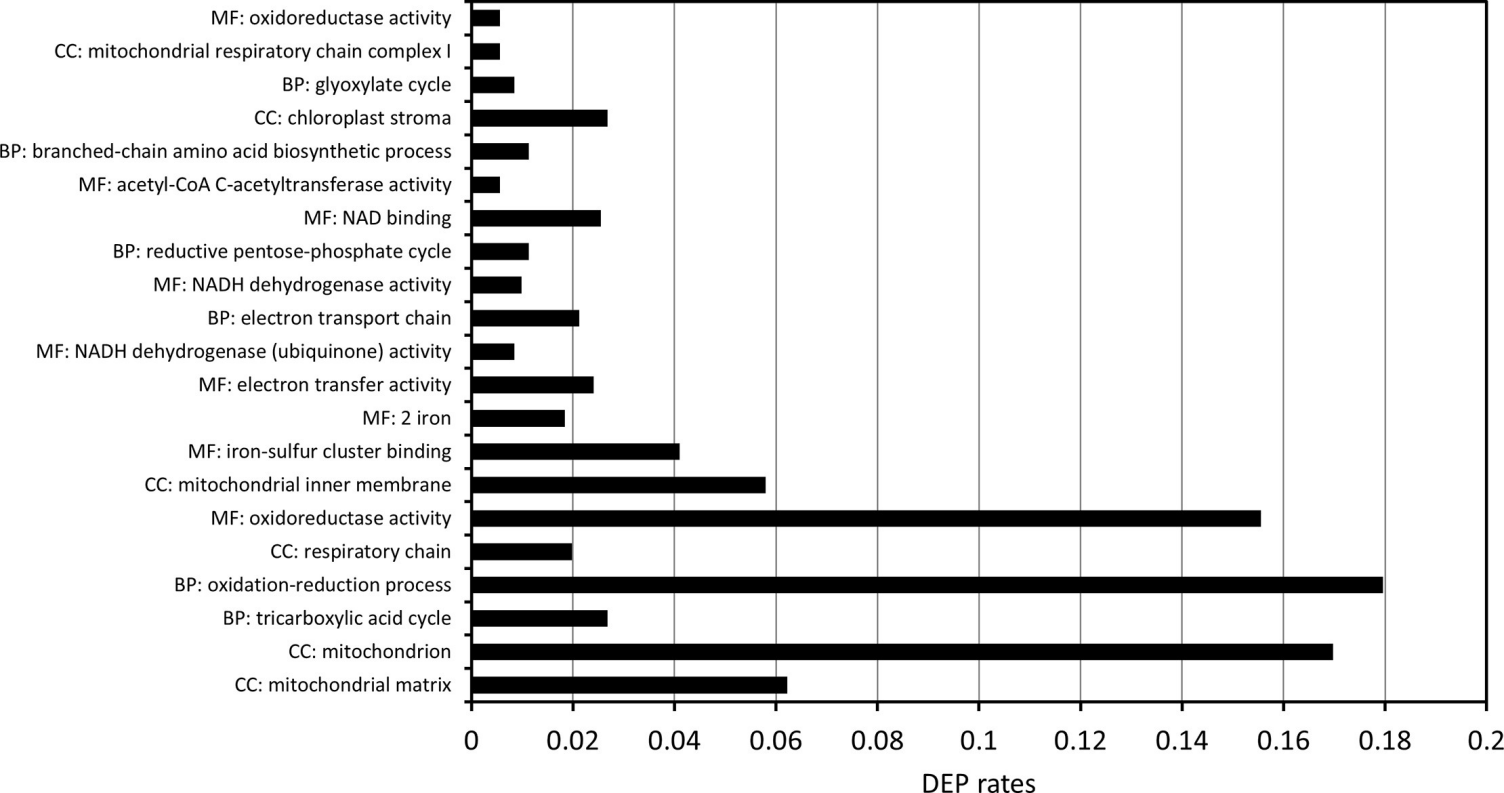
GO enrichment analysis of differentially expressed proteins. The differentially expressed protein (DEP) rate represents the ratio of number of DEPs categorized in an identical GO term to the number of all DEPs. BP, biological process; CC, cellular component; MF, molecular function.

GO enrichment analysis was performed to understand the functional categories of DEPs. The GO terms mitochondrion (GO:0005739), mitochondrial matrix (GO:0005759), and mitochondrial inner membrane (GO:0005743) as cellular component, oxidation-reduction process (GO:0055114) as biological process, and oxidoreductase activity (GO:0016491) as molecular function, showed high DEP rates (Fig 1). These results confirmed that we have successfully obtained proteins localized in mitochondria, and that dynamic expression changes in mitochondria metabolic pathways and redox reactions take place at the protein levels in response to oxygen availability. We then focused on enzymes involved in TCA cycle, glyoxylate cycle, and mitochondrial fatty acid synthesis, as these are particularly responsible for wax ester synthesis, among mitochondrial metabolic pathways.

### TCA cycle

**Fig 2:**
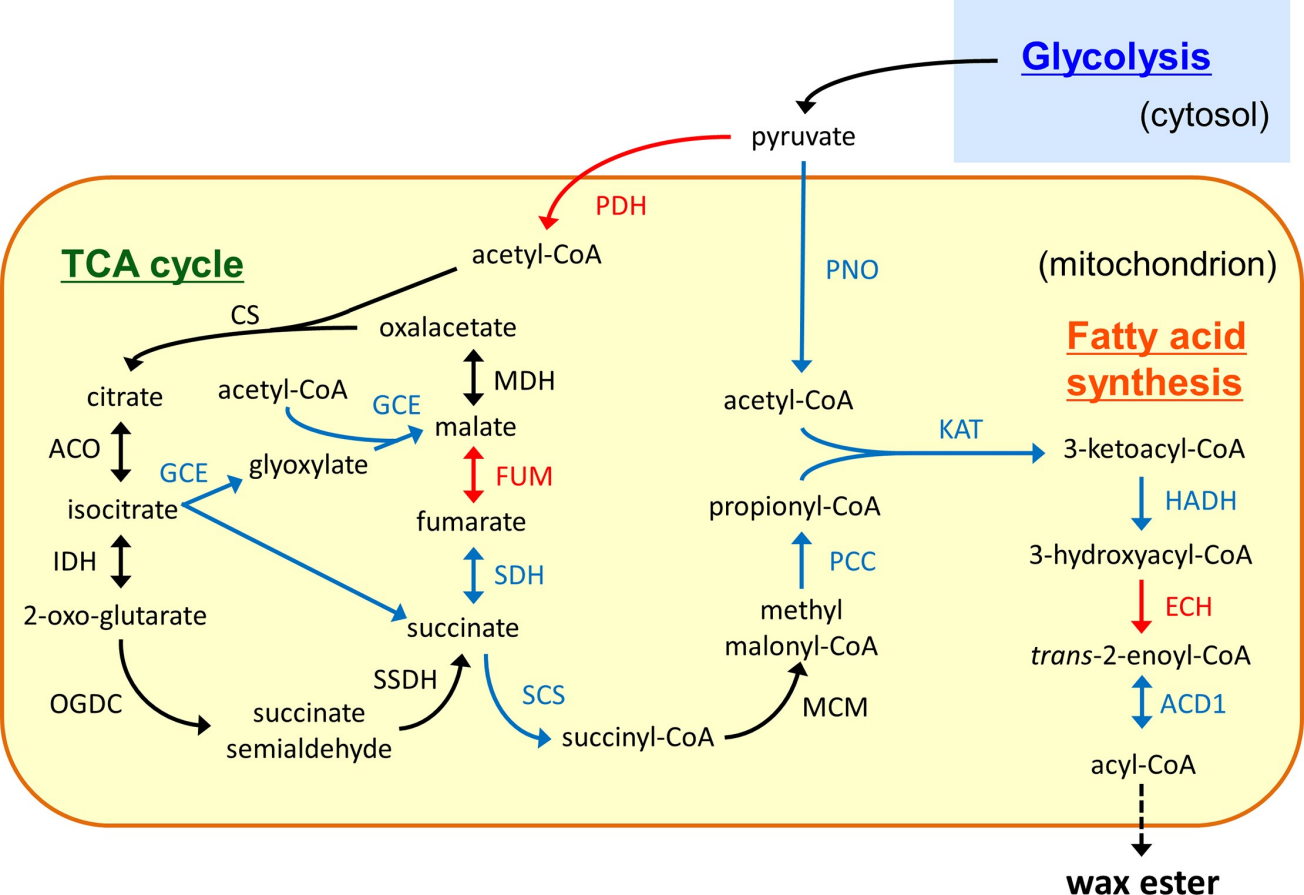
Metabolic pathways and protein levels of related enzymes in mitochondria under low-oxygen conditions. Pyruvate from glycolysis enters into the TCA cycle or fatty acid synthesis after the conversion to acetyl-CoA by PDH or PNO. The glyoxylate cycle involving GCE is the alternative route to the TCA cycle in mitochondria. Fatty acids are synthesized by a reversal of β-oxidation. Final product acyl-CoA is used for the synthesis of wax esters. Red and blue arrows represent down- and up-regulated (>1.2-fold) enzymes, respectively, under hypoxic conditions. Black arrows indicate enzymes whose expression remained unchanged upon transfer to hypoxic conditions. PDH, pyruvate dehydrogenase; CS, citrate synthase; ACO, aconitate hydratase; IDH, isocitrate dehydrogenase; OGDC, 2-oxoglutarate decarboxylase; SSDH, succinate-semialdehyde dehydrogenase; SDH, succinate dehydrogenase; FUM, fumarate hydratase; MDH, malate dehydrogenase; GCE, glyoxylate cycle enzyme; SCS, succinyl-CoA synthetase; MCM, methylmalonyl-CoA mutase; PCC, propionyl-CoA carboxylase; PNO, pyruvate:NADP^+^ oxidoreductase; KAT, 3-ketoacyl-CoA thiolase; HADH, 3-hydroxyacyl-CoA dehydrogenase; ECH, enoyl-CoA hydratase; TER, *trans*-2-enoyl-CoA reductase; ACD, acyl-CoA dehydrogenase.

The relative protein levels (hypoxic/aerobic) of enzymes involved in the TCA cycle, glyoxylate cycle, and mitochondrial fatty acid synthesis are summarized in Table 1 and their pathways are illustrated in Fig 2. The pyruvate dehydrogenase (PDH) complex plays a critical role in aerobic cell growth as a supplier of acetyl-CoA to the TCA cycle [8]. However, under low-oxygen conditions, acetyl-CoA is generated with a different enzyme PNO [4]. This was supported by the fact that the relative protein levels of PDH E1 α and β subunits decreased under hypoxic conditions (0.81- and 0.77-fold, respectively), whereas those of PNO increased, as described below (Table 1). A previous proteomic approach using two-dimensional gel electrophoresis and MS analysis showed a similar trend [18]. Incidentally, the downregulation of PDH under anoxia has been reported at transcript and protein levels in rice plants [19]. Further observations in other species, especially in protozoa and algae, are needed to evaluate whether it is a common phenomenon or not.

**Table 1:** List of identified enzymes related in wax ester synthesis.

Pathway	Annotation	Abbreviation	Sequence ID	UniProt ID	Normalized abundances		Relative protein levels	ANOVA (p)
					Aerobic	Hypoxic	(hypoxic/aerobic)	
TCA cycle	pyruvate dehydrogenase	PDH	comp26715 (α subunit)	Q6KCM1_EUGGR	1.19E+06	9.61E+05	0.81	0.0214
			comp21099 (β subunit)	—	7.05E+05	5.44E+05	0.77	0.0128
	citrate synthase	CS	comp21347	—	1.95E+06	1.90E+06	0.97	0.0002
	aconitate hydratase	ACO	comp33122	—	6.41E+06	6.13E+06	0.96	0.0862
			comp33115	—	8.52E+04	7.22E+04	0.85	0.0166
	isocitrate dehydrogenase	IDH	comp18912	—	1.06E+06	1.04E+06	0.98	0.1076
	2-oxoglutarate decarboxylase	OGDC	comp36901	A0A2Z5WHY1_EUGGR	3.65E+06	3.13E+06	0.86	0.0564
	succinate-semialdehyde dehydrogenase	SSDH	comp35901	—	3.06E+06	2.71E+06	0.89	0.0107
	succinate dehydrogenase	SDH	comp31850 (flavoprotein subunit)	—	1.96E+06	1.97E+06	1.01	0.6987
			comp8218 (iron-sulfur subunit)	—	3.11E+05	3.77E+05	1.21	0.0567
			comp36715 (iron-sulfur subunit)	—	2.56E+05	2.62E+05	1.02	0.1493
	fumarate hydratase	FUM	comp26115	—	1.27E+06	1.06E+06	0.83	0.0818
	malate dehydrogenase	MDH	comp36596 (NADP+)	—	5.25E+06	8.97E+06	1.71	0.0035
			comp30054 (NAD+)	—	2.83E+06	3.12E+06	1.10	0.9307
			comp22857 (NAD+)	—	3.56E+06	3.23E+06	0.91	0.1925
			comp20364 (NAD+)	—	1.12E+06	9.17E+05	0.82	0.0085
Glyoxylate cycle	malate synthase-isocitrate lyase	GCE	comp28766	Q8LPA6_EUGGR	2.46E+07	3.31E+07	1.35	0.0477
Fatty acid synthesis	succinyl-CoA synthetase	SCS	comp22673 (α subunit)	—	2.70E+05	3.22E+05	1.19	0.0260
			comp30619 (β subunit)	—	3.02E+05	4.15E+05	1.37	0.0153
	methylmalonyl-CoA mutase	MCM	comp28333	B7XBM0_EUGGR	9.71E+05	9.23E+05	0.95	0.1406
	propionyl-CoA carboxylase	PCC	comp29306 (α subunit)	—	9.90E+05	1.34E+06	1.35	0.0179
			comp36371 (β subunit)	—	4.61E+05	6.79E+05	1.47	0.0126
	pyruvate:NADP+ oxidoreductase	PNO	comp12747	PNO_EUGGR	1.54E+07	2.58E+07	1.68	0.0072
	3-ketoacyl-CoA thiolase	KAT	comp36638 (KAT1)	—	2.55E+06	3.45E+06	1.35	0.2977
			comp17496 (KAT2)	—	1.86E+06	2.29E+06	1.23	0.0158
			comp17821 (KAT3)	—	8.93E+06	1.06E+07	1.19	0.1095
			comp22846 (KAT6)	A0A0F7R290_EUGGR	2.95E+05	1.22E+05	0.41	0.0552
	3-hydroxyacyl-CoA dehydrogenase	HADH	comp25049	Q84T13_EUGGR	2.70E+06	3.46E+06	1.28	0.0923
			comp36568	—	1.23E+06	1.47E+06	1.20	0.0524
	enoyl-CoA hydratase	ECH	comp20039	—	8.10E+05	6.70E+05	0.83	0.0058
	*trans*-2-enoyl-CoA reductase	TER	comp34527 (TER1)	TER_EUGGR	9.52E+04	1.71E+05	1.80	0.0065
	acyl-CoA dehydrogenase	ACD	comp29154 (ACD1)	—	6.29E+06	8.91E+06	1.42	0.0161

Values are the mean (n = 3).

Enzyme 2-oxoglutarate decarboxylase (OGDC), which is a unique enzyme involved in the *E*. *gracilis*-specific pathway of the TCA cycle, catalyzes the conversion of 2-oxoglutarate to succinate semialdehyde and is essential for aerobic growth [8]. Succinate semialdehyde is then converted into succinate by succinate semialdehyde dehydrogenase (SSDH). *E*. *gracilis* lacks 2-oxoglutarate dehydrogenase but shows an alternative route composed of OGDC and SSDH in the TCA cycle [8,20]. Succinate is converted to propionyl-CoA by a sequential catalysis of succinyl-CoA synthetase (SCS), methylmalonyl-CoA mutase, and propionyl-CoA carboxylase (PCC) to provide C3 donor for odd-chain fatty acid synthesis. The relative protein levels of the SCS β subunit and the PCC α and β subunits increased by 1.35- to 1.47-fold under hypoxic conditions (Table 1), suggesting that upregulation of these enzymes contributed to the promotion of odd-chain fatty acid synthesis.

The NADP^+^-dependent malic enzyme (malate dehydrogenase, MDH) plays an important role in electron transport from the cytosol to the mitochondria across the mitochondrial membrane [21]. The relative protein expression level of NADP^+^-dependent malic enzyme (comp36596) increased 1.71-fold under hypoxic conditions (Table 1). This agreed with previous report of increased NADP^+^-dependent malic enzyme activity in *E*. *gracilis* under anaerobiosis [22]. Therefore, the upregulation of the NADP^+^-dependent malic enzyme may contribute to the electron supply required for wax ester synthesis.

### Glyoxylate cycle

The glyoxylate cycle is important for gluconeogenesis and the conversion of wax esters to paramylon in *E*. *gracilis* when the cells are transferred from hypoxic to aerobic conditions [23]. The glyoxylate cycle enzyme (GCE), which is a unique bifunctional enzyme having isocitrate lyase and malate synthase activities, is a key enzyme in the glyoxylate cycle of *E*. *gracilis* [24]. Interestingly, the relative protein level of GCE was 1.35-fold higher under hypoxic conditions, despite its role in wax-ester degradation (Table 1). Because the GCE reaction produces succinate from isocitrate without carbon loss and, then, provides propionyl-CoA, it might be possible that this enzyme is involved in the synthesis of odd-chain fatty acids in response to low-oxygen conditions. This could be supported by the fact that *E*. *gracilis* accumulated a large amount of succinate under low-oxygen and dark conditions [14,25].

### Mitochondrial fatty acid synthesis

PNO catalyzes the oxidative decarboxylation of pyruvate to acetyl-CoA in hypoxic mitochondria. A recent study showed that suppression of *PNO* expression markedly inhibits wax-ester production under anaerobic conditions in *E*. *gracilis* SM-ZK, suggesting that PNO plays a critical role in hypoxic fatty acid synthesis in mitochondria [8]. As shown in Table 1, proteomic analysis showed that the relative protein level of PNO increased 1.68-fold under hypoxic conditions, thus underlining the importance of this enzyme in mitochondrial fatty acid synthesis. In contrast, our previous transcriptomic analysis using wild-type Z strain showed that the *PNO* transcript level decreased 0.60-fold upon hypoxic treatment for 24h [15]. This discrepancy suggests that PNO expression is regulated post-transcriptionally. For the regulation of gene expression in *E*. *gracilis* in response to various environmental conditions, many studies have confirmed that post-transcriptional control is more important than transcriptional control [18, 26–36]. Thus, a similar mechanism might apply for PNO regulation under low-oxygen conditions.

KAT is a condensing enzyme of mitochondrial fatty-acid synthesis, and *E*. *gracilis* contains at least six predicted KAT isoforms (KAT1-6). It has been considered that KAT1, KAT2, and KAT3 are major 3-ketoacyl-CoA thiolases involved in mitochondrial fatty-acid synthesis in *E*. *gracilis*, and Nakazawa et al. (2015) [9] proposed that KAT1 and KAT2 catalyze medium- and long-chain acyl-CoA synthesis, while KAT3 catalyzes short- and medium-chain acyl-CoA synthesis. Proteomic analysis indicated that the relative protein levels of KAT1, KAT2, and KAT3 increased 1.35-, 1.23-, 1.19-fold under hypoxic conditions. In contrast, the relative protein levels of KAT6 decreased 0.78-fold under hypoxic conditions. As the knockdown of KAT6 was found to not affect wax ester production under anaerobic conditions [9], this enzyme may not be involved in the process of wax ester fermentation. Neither KAT4 nor KAT5 isoforms were identified in mitochondrial proteomic analysis, strongly supporting that their localization is not in mitochondria.

Previously, TER was believed to catalyze the final step of fatty acid synthesis from enoyl-CoA to acyl-CoA and enable malonyl-CoA independent fatty acid synthesis in *E*. *gracilis* mitochondria [6,10]. However, our recent study showed that TER1 is dispensable for wax ester production, although it is still necessary for chloroplast development [11]. Moreover, it has been suggested that acyl-CoA dehydrogenase isoform 1 (ACD1) catalyzes the reduction of *trans*-2-enoyl-CoA in *E*. *gracilis* cells, although knockdown of *ACD1* does not completely inhibit wax ester production [37]. Proteomic analysis showed that the relative protein levels of TER1 and ACD1 increased 1.80- and 1.42-fold under hypoxic conditions, respectively (Table 1). However, the normalized abundance of TER1 was very low and represented only 1.9% that of ACD1 under hypoxic conditions (Table 1). Thus, the protein expression level of TER1 is likely to be very low even under low-oxygen conditions, although it has been proved that an enzyme activity against a short chain CoA (crotonyl-CoA) derived from TER1 is dominant based on results of our previous TER1 gene knockdown experiment [11].

### Proteolysis

The proteins comp29057, 35721, 28384, 36301, and 28283, which are annotated as cysteine proteases, including cathepsins, were identified twice in three-independent analyses, and their relative protein levels decreased 0.31–0.38-fold under hypoxic conditions (Table 2). One of these, comp29057 protein was actually expected to be localized in mitochondria based on TargetP prediction (Table 2). Cathepsins are ubiquitous proteases essential for the survival of *Trypanosoma brucei*, a protozoan parasite phylogenetically close to *E*. *gracilis* [38]. It has been reported that the transcript levels of proteases, including those of cysteine protease, increase during anoxia acclimation in *Chlamydomonas reinhardtii*, suggesting a correlation between protease expression and low-oxygen treatment [39]. Although the physiological role of cathepsins in hypoxic metabolism is unknown, it is possible that these enzymes regulate the protein levels of the fatty acid synthesis-related enzymes in response to low-oxygen conditions.

**Table 2:** List of identified cysteine proteases.

Annotation	Sequence ID	TargetP prediction	Normalized Abundances		Relative protein levels
			Aerobic	Hypoxic	(hypoxic/aerobic)
Cathepsin	comp29057	Mitochondrion	6.08E+04	2.48E+04	0.41
	comp35721	―	4.97E+04	2.08E+04	0.42
	comp28384	Secretory	1.86E+05	5.74E+04	0.31
	comp36301	Secretory	6.64E+03	2.49E+03	0.38
Cysteine protease	comp28283	Secretory	2.90E+05	9.67E+04	0.33

These cysteine proteases were identified twice among three independent experiments. TargetP program (http://www.cbs.dtu.dk/services/TargetP/) was used to predict the subcellular localization of identified cysteine proteases. Values are the mean (n = 2).

## Conclusions

Intact mitochondria were isolated from *E*. *gracilis* under aerobic and hypoxic conditions and used for shotgun proteomic analysis to understand the regulatory mechanism of wax ester synthesis in this organism. Our study demonstrated that the relative protein levels of enzymes involved in the TCA cycle (MDH), the glyoxylate cycle (GCE), and mitochondrial fatty-acid synthesis (SCS, PCC, PNO, KAT1, KAT2, HADHs, and ACD1) all increased (1.20- to 1.71-fold) under hypoxic conditions, suggesting that hypoxic mitochondrial metabolism for wax ester synthesis is activated by the upregulation of enzymes involved at the protein level, but not at the transcript level [15]. Our mitochondrial proteome analysis provides new insight into the regulatory mechanism underlying the metabolic shift under hypoxic conditions. It might be possible to speculate that an unknown mechanism which facilitates translocation of precursor enzymes into mitochondria in response to low-oxygen condition is functioning. Or, a small RNA molecule might regulate translational levels of some particular proteins as recently reported in *Trypanosoma brucei* [40]. It should be noted, however, that the rate of increase in mitochondrial proteins was less than double, which is probably not enough to explain the drastic metabolic shift. Thus, the process of wax ester fermentation is likely to be regulated at the post-translational level as well as the translational level. In fact, a recent reverse genetic analysis of starch degradation-related kinase genes (*STD1* and *STD2*) suggested the possibility of the protein phosphorylation-mediated regulation of wax ester production in *E*. *gracilis* [41]. A functional proteomic approach, such as a phospho-proteome analysis, will help us to expand our understanding of the regulatory mechanism of wax ester production in *E*. *gracilis* in future studies.

## Supporting information

S1 Table List of differentially expressed proteins in mitochondria isolated from *E. gracilis* cells treated with aerobic and hypoxic conditions. (XLSX)
